# Osteosynthesis-associated infection in maxillofacial surgery by bacterial biofilms: a retrospective cohort study of 11 years

**DOI:** 10.1007/s00784-023-05059-2

**Published:** 2023-05-13

**Authors:** Matthias Zirk, Waldemar Markewitsch, Franziska Peters, Nadja Kröger, Max-Philipp Lentzen, Joachim E. Zoeller, Max Zinser

**Affiliations:** 1 Department for Oral and Craniomaxillofacial and Plastic Surgery, University of Cologne, Kerpener Strasse 62, 50931 Cologne, Germany; 2 Department of Plastic, Reconstructive and Aesthetic Surgery, Faculty of Medicine and University Hospital Cologne, University of Cologne, Cologne, Germany; 3 Department of Dermatology, University of Cologne, Cologne, Germany; 4 Institute for Medical Microbiology, Immunology and Hygiene, University Hospital of Cologne, Cologne, Germany

**Keywords:** Oral biofilms, Osteosynthesis-associated infections, Oral infections

## Abstract

**Objectives:**

The aim of this retrospective cohort study was to determine risk factors for osteosynthesis-associated infections (OAI) with subsequent necessity of implant removal in oral and maxillofacial surgery.

**Materials and methods:**

A total of 3937 records of patients who received either orthognathic, trauma, or reconstructive jaw surgery from 2009 to 2021 were screened for osteosynthetic material removal due to infection. Treatment-intervals, volume of applied osteosynthetic material, and respective surgical procedures were also assessed. Moreover, intraoperatively harvested microbial flora was cultured and subsequently identified by MALDI TOF. Bacteria were then screened for antibiotic resistance via VITEK system or, if necessary, via agar diffusion or epsilometer test. Data was analyzed utilizing SPSS statistical software. For statistical analysis of categorical variables, chi-square tests or Fisher exact tests were used. Continuous variables were compared via non-parametric tests. The level of significance for *p*-values was set at < 0.05. Descriptive analysis was also performed.

**Results:**

The lower jaw was more prone to OAI than the mid face region. Larger volumes of osteosynthetic material led to significantly more OAI, resulting in reconstruction plates bearing the highest risk for OAI especially when compared to small-volume mini-plates frequently applied in trauma surgery. Among OAI associated with implant volumes smaller than 1500 mm^3^, the detection of *Streptococcus spp., Prevotella spp., Staphylococcus spp.*, and *Veillonella spp*. was significantly elevated, whereas implant volumes larger than 1500 mm^3^ showed a significant increase of *Enterococcus faecalis*, *Proteus mirabilis* and *Pseudomonas aeruginosa*. High susceptibility rates (87.7–95.7%) were documented for 2nd- and 3rd-generation cephalosporines and piperacillin/tazobactam.

**Conclusion:**

High material load and lower jaw reconstruction bear the greatest risks for OAI. When working with large volume osteosynthetic implants, gram-negative pathogens must be considered when choosing an appropriate antibiotic regime. Suitable antibiotics include, e.g., piperacillin/tazobactam and 3rd-generation cephalosporines.

**Clinical relevance:**

Osteosynthetic material utilized in reconstructive procedures of the lower jaw may be colonized with drug-resistant biofilms.

## Introduction

Internal osteosynthesis has been proven a substantial aid within oral and maxillofacial surgery, thus eliminating the necessity for external fixation devices [22]. Titanium plate fixation remains common among trauma and oncologic procedures. Reconstruction is therefore regularly achieved via rigid fixation, e.g., a single locking titanium reconstruction plate (e.g., 2.0, 2.5, or 2.7 mm plate profiles). Sometimes, titanium mini-plates may also be applied for reconstructive purposes if in accordance with the surgeon’s experience and preference [14]. Furthermore, internal fixation utilizing osteosynthetic material is also required for orthognathic surgery. Late trends in reconstructive and orthognathic surgery thus present enhanced wafers, designed and manufactured with computer assistance (CAD/CAM), as well as patient-specific saw guides for individually formed osteosynthesis plates [24]. Plate osteosynthesis in craniomaxillofacial surgery has been reported as reliable, safe, and effective in various clinical studies [3]. Handling of most anatomical landmarks remains relatively easy for experienced surgeons, although certain cases such as mandible collum fractures may become challenging [3]. Due to the low incidence of complications within the mid and upper face, asymptomatic titanium plates must not be necessarily removed. In contrast, high rates of mandibular site complications may demand routine removal of osteosynthesis plates within this area of the head [19]. Particularly within the mandible, titanium plates are not only inserted into a non-sterile field but regularly exposed to strong oral and maxillofacial forces during mastication. This may lead to infection or plate breakage [23]. While the respiratory and alimentary tracts are incised under controlled intraoperative conditions [33], a breach of the mucosal barrier leads to exposure of the wound to fluids and biofilms originating from the oral cavity [20, 33].

When inoculated, planktonic bacteria are susceptible to host defense only initially. Once bacteria pass through the damaged soft tissue and reside at the bone-implant interface, rapid multiplication takes place. As infestation increases, the infection continually spreads [9, 18]. Furthermore, bacterial biofilms colonize osteosynthetic material and bone, further leading to osteitis, bone necrosis, reduction of new bone formation, and provoked emptying of Haversian canals [9, 18]. Implant-associated biofilms are characterized throughout groups of bacteria which are thoroughly encased in extracellular matrix and thus significantly less susceptible to antimicrobial agents, especially when compared to regular planktonic bacteria [12]. Moreover, attempted phagocytosis of biofilm-associated organisms by endogenous macrophages may even cause more damage throughout the involvement of surrounding tissues [9, 13]. Thus, material removal in the case of recurrent bacterial infection seems inevitable [8, 26].

The aim of this retrospective cohort study was to individually evaluate the volume of osteosynthetic material, its location, and the respective operative procedures within the jaw and face as risk factors for early implant removal due to bacterial colonization. Harvested pathogens were further investigated with regard to antibiotic susceptibilities as documented within the patient records.

## Material and methods

A retrospective cohort study of 11 years was carried out from 2009 to 2020 and was accordingly approved by the University Clinic of Colognes Ethic Committee (22–1093-retro). Medical documents, surgical documentations, and postoperative radiographic images of patients (*n* = 3937) that had received corresponding surgeries at the University of Cologne were thus reviewed and investigated. Ages ranged from 18 to 84 years with a mean of 54 (± 18.7 SD) years. No patients under the age of 18 were included in this study.

A total of 92 patients with surgical site infections associated with osteosynthetic material, in-hospital treatment, and subsequent implant removal were identified and included in the cohort. The diagnosis of surgical site infection was based on the following criteria originating from the Center of Disease Control: https://www.cdc.gov/nhsn/pdfs/pscmanual/9pscssicurrent.pdf

Inclusion criteria encompassed the installation of osteosynthetic plates for the treatment of mandibular/mid face fractures, reconstructions, and osteotomies.

Exclusion criteria were defined as osteosynthetic devices other than plates and osteosynthetic material applied outside the jaw and face area. Patients that had undergone surgical removal of osteosynthesis plates elsewhere were also excluded.

Documented data of the surgical interventions’ consumables and postoperative X-ray images were reviewed and compared in order to further specify the properties of the utilized implant. Particular attention was paid to the number of screws and potential modification of plates, as, e.g., plate shortening.

Calculation of screw and plate volumes was carried out with respect to catalogue data within the manufacturers’ manual. Product information on thickness, length, diameter, and visualization on a 1:1 scale were utilized in order to idealize volume by applying geometric calculation formulas.

By dividing the implant surface into different defined geometrical areas, the total surface area of a plate was preliminarily calculated by adding the results of corresponding area formulas, e.g., the area formula for triangles (*A* = g⋅*h*/2), the circle segment formula (*A* = *r*2/2⋅(*α*⋅*π*/180° − sin (*α*))), the parabolic segment formulas (*A* = 1/3⋅*h*⋅*a* and *A* = 2/3⋅*h*⋅*a*), and the area formula for rectangles (*A* = length⋅width). To further address the volume specifics of a plate, the previously calculated total area was then multiplied by the thickness of the plate. In order to simplify geometric scaling, 1:1 representations were scanned and subsequently measured in an enlarged version. The determined total values were then converted back into the real size via the rule of three. Screw volumes were calculated in a likewise manner.

**Fig. 1:**
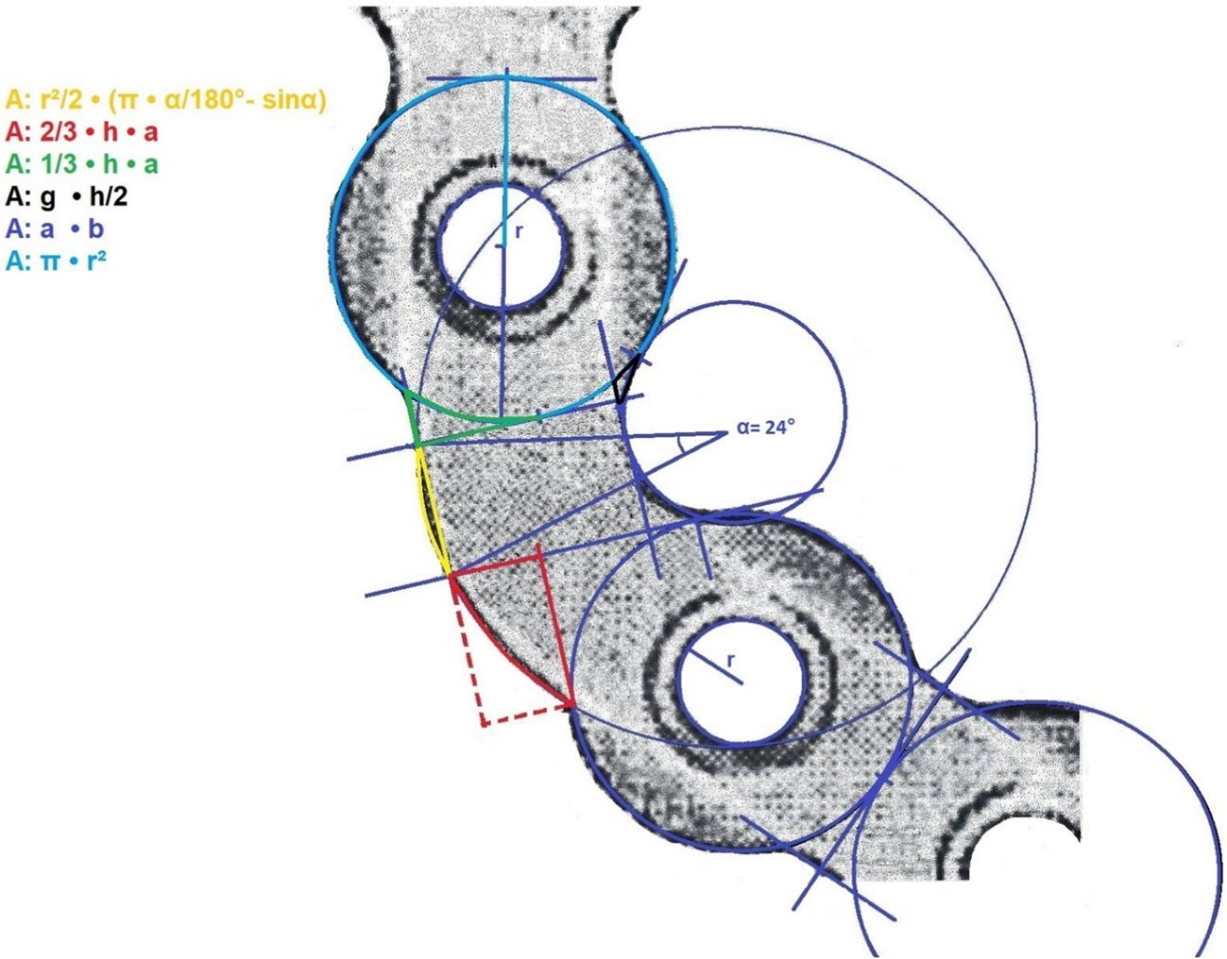
Illustration of a 2.7 reconstruction plate and its respective measurements for calculation of the total volume (manufacturer: KLS Martin GmbH & Co. KG, 2019, Tuttlingen, Germany)

Figure 1 shows calculation of volume.

Surgical site infections were intraoperatively sampled and forwarded to the Institute for Medical Microbiology at the university clinic for further analysis. Microorganisms were cultivated and species identification was carried out via matrix-assisted laser desorption ionization (MALID) in combination with time-of-flight analysis (TOF) of released ions for mass spectometry (MALDI-TOF). In accordance with the guidelines of the European Committee for Antimicrobial Susceptibility Testing (EUCAST), susceptibility tests were then carried out via VITEK (Biomerieux) and completed by agar diffusion or epsilometer tests, if necessary. Results were then back-reported to the Department of Oral and Maxillofacial Surgery for clinical reevaluation. All reports comprising human pathogenic bacteria were considered in this study. The reports on antimicrobial resistance patterns were evaluated with regard to frequently applied antibiotics.

All data was pseudonymized when extracted from the clinics’ database and analyzed via SPSS statistical software (SPSS version 27.0; IBM, Munich, Germany).

First, descriptive analysis was performed. For statistical analysis of proportions across levels of categorical variables, either chi-square tests or Fisher exact tests were used.

Continuous variables were compared via non-parametric Kruskal–Wallis and Mann–Whitney-U or Wilcoxon signed-rank tests. The Bonferroni or GT2 Hochberg adjustment was employed to counteract the problem of multiple comparisons. The level of significance for *p*-values was set at < 0.05.

## Results

**Fig. 2:**
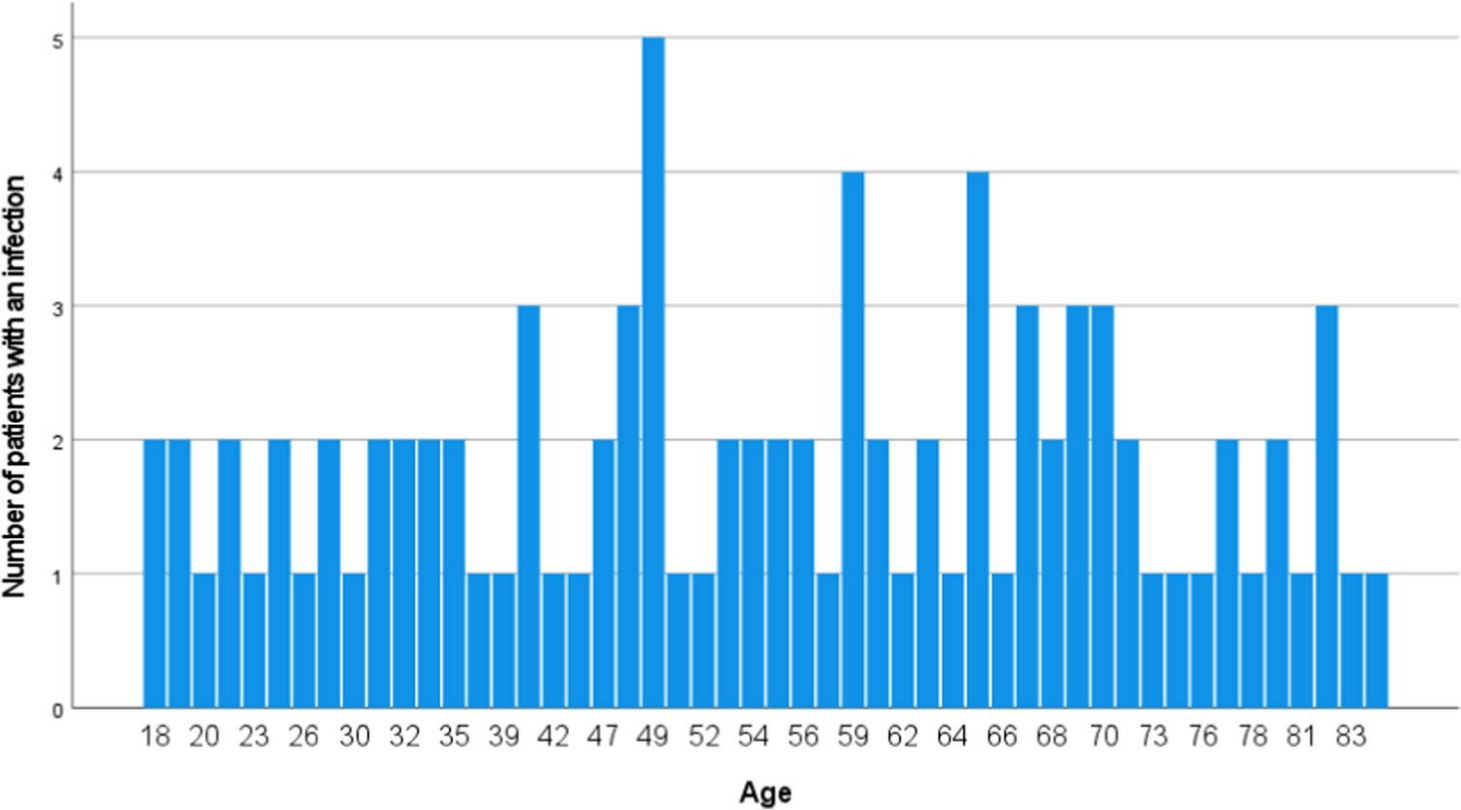
Distribution of age

In total, 92 out of 3937 (2.4%) patients with built-in osteosynthetic material presented a respective surgical site infection. Removal of bacterially colonized material was more common among males (*n* = 62; 67.4%) than females (*n* = 30; 32.6%). Ages ranged from 18 to 84 years, resulting in an average age of 53 with a standard deviation of ± 18.7 SD, as illustrated in Fig. 2.

The lower jaw was affected significantly more by surgical site infection (SSI) and subsequent plate removal than the mid face area (*p* < 0.05). The anatomic variability of surgical sites with colonized osteosynthetic plates is displayed in Table 1.

**Table 1:** List of the various locations of plate removal. The lower jaw was significantly more prone to SSI, *p* ≤ 0.05

Localization	*n* (%)
Left lower jaw	36 (39.1%)
Right lower jaw	34 (37%)
Both sides lower jaw	9 (9.8%)
Left mid face	6 (6.5%)
Midline of lower jaw	3 (3.3%)
Right mid face	3 (3.3%)
Mid face, left + right	1 (1.1%)
Total lower jaw	82 (89.1%)
Total, mid face	10 (10.9%)
Total	92 (100%)

The median of plate insertion and plate removal was 141 days (interquartile range of 233 days with 48 days for the 25th percentile and 280.5 days for the 75th percentile). A total of 128 bacterially colonized plates were removed; 2.7/2.3 reconstruction plates represent 2 different plate types. The numbers represent the diameter of the respective plate holes. A detailed overview of all removed plates is given in Table 2.

**Table 2:** The removed and respective total of previously inserted osteosynthetic plates of various sizes are listed

Osteosynthesis	*n* (%)	Total of osteosynthesis (*n*)
2.7 Reconstruction plate	26 (20.3%)	365
2.3 Reconstruction plate	8 (6.3%)	252
Reconstruction plate (other origin)	2 (1.6%)	
Mini plate (long)	32 (25%)	3074
Mini plate (short)	22 (17.2%)	2066
BSSO plates	14 (10.9%)	898
Micro-plate (long)	15 (11.7%)	3384
Micro-plate (short)	4 (3.1%)	2287
Others	5 (3,9%)	328
Total	128 (100%)	

**Fig. 3:**
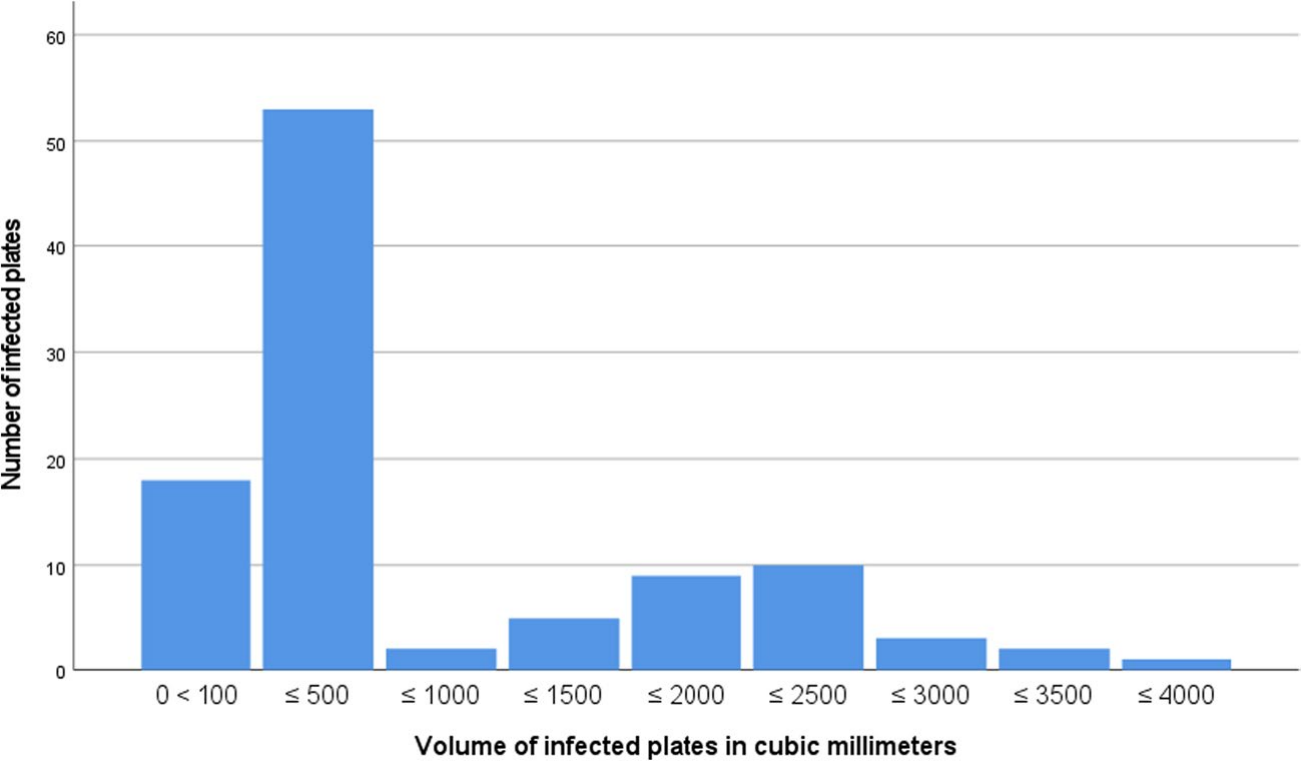
Numbers and the volumes of removed implants (plates plus screws)

Analysis of the inserted osteosynthetic materials (plate plus fixation-screws) revealed that the bulk of removed plates amounted to volumes up to 500 mm^3^. But aforementioned was also more frequently inserted (*n* = 2065 surgical sites). They, namely, comprised mini plates and bilateral sagittal split osteotomy (BSSO) plates. Plates utilized for reconstructive procedures amounted to volumes larger than 500 mm^3^. Calculated implant volumes are illustrated in Fig. 3 and Table 3.

**Table 3:** Comparison of infected and non-infected plates

Osteosynthesis	Volume in mm^3^	Infected (*n*)	Non infected (*n*)
	≤ 2000	9	126
2.7 Reconstr. plate	≤ 3000	12	182
	≤ 4000	3	31
	≤ 1000	2	65
2.3 Reconstr. plate	≤ 2000	5	134
	≤ 3000	1	45
Mini-plates, short	≤ 120	14	2044
Mini-plates, long	≤ 500	26	3042
BSSO plates	< 304, 94	14	884
Micro-plates	100	17	5652

**Fig. 4:**
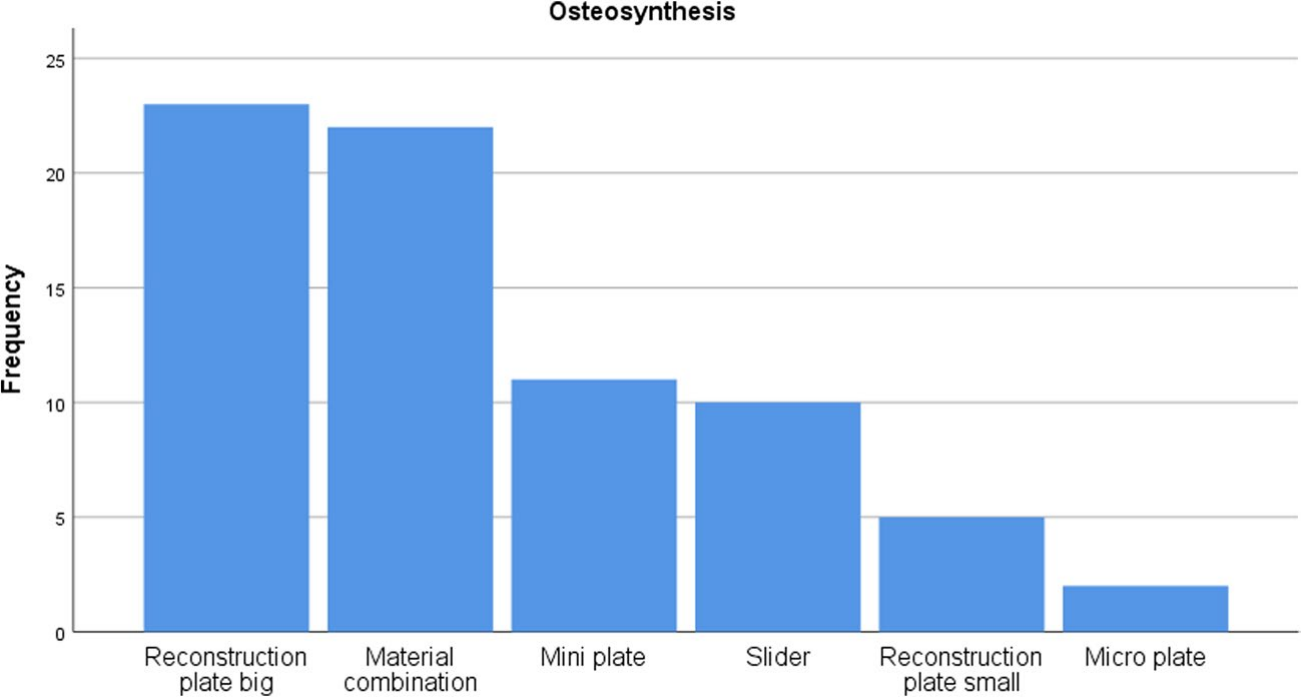
Numbers and types of removed osteosynthetic material

The largest implant volumes were associated with reconstruction plates while being inserted less often than mini-plates or BSSO plates. In relation to the total number of each type of osteosynthetic material, larger reconstruction plates were significantly more prone to infections (*p* 0.05), as seen in Fig. 4.

The study’s cohort consisted of 557 lower jaw reconstructions with 39 surgical site infections (7%). Furthermore, 1610 lower jaw fractures were treated via osteosynthesis with mini-plates, with a total of 31 experiencing SSI (1.9%). In addition, 455 patients underwent corrective jaw surgery within the lower jaw; among these, 15 presented with a SSI (3.3%). For mid face fractures, 7 out 1315 patients experienced a SSI (0.5%).

With regards to SSI, results differed significantly between the various types of osteosynthetic material: reconstruction plate > BSSO-plate > mini-plate > mid face micro-plate (Table 4).

**Table 4:** Types of surgery

Time span (2009–2020)	Fracture treatment, lower jaw	Fracture treatment, mid face	Corrective lower jaw surgeries	Jaw reconstructions
Total	1610	1315	455	557
Infections	31 (1.9%)	7 (0.5%)	15 (3.3%)	39 (7%)

Polymicrobial flora of the colonized osteosynthetic materials consisted mainly of facultative anaerobic gram-positive and gram-negative bacteria, as displayed in Tables 5 and 6. Streptococci of the Viridans and the non-Viridans group were isolated most frequently; among Prevotella species, Staphylococci, Fusobacterium species, *Escherichia coli*, and Veillonella species also detected very frequently, as seen in Table 5.

**Table 5:** Pathogens harvested from SSI in relation to osteosynthetic material

Pathogens	*n* (%)
*Streptococcus spp*.	22 (16.7%)
*Prevotella spp.*	17 (12.9%)
*Staphylococcus spp.*	16 (12.1%)
*Fusobacterium spp.*	9 (6.8%)
*Veillonella spp.*	8 (6.1%)
*Escherichia coli*	7 (5.3%)
*Parvimonas micra*	7 (5.3%)
*Pseudomonas aeruginosa*	4 (3%)
*Enterococcus faecalis*	4 (3%)
Klebsiella spp.	4 (3%)
*Proteus mirabilis*	3 (2.3%)
Bacteroides spp.	3 (2.3%)
*Enterobacter spp.*	3 (2.3%)
Others	25 (18.9%)
Total	132 (100%)

**Table 6:** Preferred milieu and gram staining of pathogens

Pathogens	*n* (%)
Facultative anaerobic gram-positive	42 (31.8%)
Anaerobic gram-negative	38 (28.8%)
Facultative anaerobic gram-negative	29 (22%)
Anaerobic gram-positive	16 (12.1%)
Facultative aerobic gram-negative	4 (3%)
Aerobic gram-negative	3 (2.3%)
Total	132 (100%)

A total of 59 patients presented with usable microbiological analyses, whereas 61% (*n* = 36) suffered from a polymicrobial infection and 39% (*n* = 23) from a monomicrobial infection.

Bacterial species such as *Streptococcus spp.*, *Prevotella spp.*, *Staphylococcus spp.*, and *Veillonella spp.* were associated with low volumes of osteosynthetic material, whereas *E. faecalis*, *P. mirabilis*, and *P. aeruginosa* were associated with larger material volumes.

Multidrug resistance was merely confirmed for two of the cohort’s pathogens. Susceptibility testing revealed one *Staphylococcus aureus* as MRSA and one *P. aeruginosa* as 4-MRGN. With regard to the overall resistance pattern, the highest susceptibility rates were identified for cefotaxime and imipenem, as seen in Table 7. Similar rates were also identified for 2nd- and 3rd-generation cephalosporines and carbapenems.

**Table 7:** Antibiotic resistance patterns

Antibiotics	Number of tested bacteria	Susceptible bacteria	Resistant bacteria	Overall susceptibility rate%
Cefotaxime^(3rd generation)^	47	45	2	95.7
Imipenem	61	58	3	95.1
Meropenem	31	29	9	93.5
Co-Trimoxazole	45	41	4	91.1
Ceftazidime^(3rd generation)^	28	25	3	89.3
Cefuroxime^(2nd generation)^	54	49	5	88.9
Piperacillin/tazobactam	65	57	8	87.7
Gentamicin	45	40	5	86.7
Tigecyclin	38	32	6	84.2
Moxifloxacin	34	28	6	82.4
Tetracycline	32	26	6	81.3
Piperacillin	45	34	11	75.5
Ampicillin/sulbactam	68	51	17	75
Ampicillin	53	34	19	64.2
Erythromycin	22	14	8	63.6
Clindamycin	39	24	15	61.5
Ciprofloxacin	65	39	26	58.5

For ß-lactam antibiotics with or without ß-lactamase inhibitor, significantly higher susceptibility was noted for the combination piperacillin/tazobactam rather than piperacillin or ampicillin (*p* < 0.05; Table 7) by itself. Susceptibility was also significantly higher in comparison to ampicillin/sulbactam (*p* < 0.05; Table 7).

Adequate susceptibility rates were also identified for co-trimoxazole and gentamicin if bacteria were within their respective therapeutic index. About half of all the isolates tested for clindamycin, erythromycin, and ciprofloxacin revealed resistances toward these antibiotics (Table 7).

Further risk factors investigated in our study comprised multimorbidity, teeth in the fracture gap, diabetes mellitus, chronic renal insufficiency, hepatitis C, and alcohol, nicotine, and substance dependence, as well as malignant tumorous diseases, as seen in Table 8.

**Table 8:** Risk factors based on general health

Number of risk factors for infection in the area of osteosynthesis (*n*)	Number of patients with postoperative infection *n* (%)
0	18 (19.6%)
1	41 (44.6%)
2	12 (13%)
3	17 (18.5%)
4	3 (3.3%)
5	1 (1.1%)
Total	92 (100%)

## Discussion

In this study, infections of osteosynthetic surgical sites with subsequent necessity of implant removal were investigated. The occurrence of this clinical entity, namely, surgical site infection after osteosynthesis, has been referred to as osteosynthesis-associated infection (OAI) within earlier reports [9]. Early removal of bacterially colonized osteosynthetic material may eventually lead to impaired fracture stability with inadequate and prolonged healing, which in turn may be associated with prolonged hospitalization and an increase in healthcare costs [21]. Besides consequent implant removal, the traditional treatment of osteosynthesis-associated infections among oral and maxillofacial surgeons (OMFS) consisted of tooth extraction, sequestrectomy, drainage, and immobilization of the fracture with either maxillomandibular fixation (MMF), splints, or comparable devices and concomitant antibiotic treatment [1]. Some surgeons even chose to replace the internal osteosynthetic material with more rigid internal osteosynthetic fixation and bone grafts once the source of infection was eliminated [1, 16] instead of applying the method of external splinting. Nevertheless, hardware removal remains inevitable when signs of osteomyelitis are present and bone does not heal [6]. Despite various studies on osteomyelitis among OMFS, up until now, none have thoroughly investigated the risk of implant volumes in relation to osteosynthesis-associated infections (OAI).

Within this investigation, 92 out of 3937 (2.4%) were diagnosed with an OAI. According to previous literature, rates of infection ranged from 2.7 to 26.8% and osteosynthesis removal rates ranged from 2.3 to 28.1% [6]. Also, infections related to cranio-maxillofacial surgery (CMF) hardware do not appear nearly as often as those related to osteosynthesis of extremities (5–15% in CMF fractures vs. 5–50% within extremities). This phenomenon may be based on the unique anatomy of the face with its watershed blood supply, thus being less susceptible to vascular compromises when compared to extremities [6]. The blood supply of extremities depends on major vessels which may be compromised in various manners, e.g., trauma or vascular disease [6]. In contrast, relatively low rates of OAI within this study may be related to frequent postoperative wound surveillance and perioperative antibiotic regimes.

Within this study, the lower jaw was significantly more prone to infection, as seen in Table 1. In accordance with earlier reports [7, 32], the lower jaw seems to be more vulnerable to invasive bacterial infections when compared to the upper jaw or the mid face [7, 32]. A total of 31 (1.9%) out of 1610 lower jaw trauma sites experienced OAI. Slider plates used in BSSO (13/455; 3.3%) were significantly more prone to infection. Earlier reports have already determined that the type of orthognathic surgery performed qualifies as one of the major risk factors for infection in orthognathic surgery, identifying mandibular osteotomies (in bilateral sagittal split osteotomy (BSSO)) as one of the most precarious [25]. Within this cohort, plates with volumes ≤ 500 mm^3^ were most frequently applied (Fig. 3), thus also amounting to the greatest number with respect to OAI (Table 2). Nevertheless, the infection rate of mini plates utilized in lower jaw trauma surgery was the second lowest within the cohort.

The rate of mandibular reconstruction failures (reconstruction plate removal) due to plate exposure and other complications has been reported as high (50–80%) [14]. In some cases, surgeons tend to choose a soft tissue cover and reconstruction plate rather than an osteo-cutaneous flap for simple wound closure [28]. This approach may be accompanied by plate-related problems such as plate exposure and plate loosening as a consequence of bacterial biofilm colonization and the subsequent development of an OAI [15, 28]. Bacterial colonization of osteosynthetic material is a risk factor for definitive OAI. However, the onset of an OAI remains a complex interplay between the host immune defenses, the osteosynthetic material, and the pathogen [15]. Due to the lack of blood vessels on osteosynthetic materials, immune defense only has very limited access to the foreign body [31]. It has additionally been observed that immune defense cells may express decreased activity when interacting with specific osteosynthetic materials [15]. Hence, osteosynthetic materials of larger volumes are more prone to bacterial colonization and thus more susceptible to OAI, as shown in Fig. 3.

Submucosal biofilms differ from biofilms harvested from the bottom of osseous lesions, yet *Streptococcus spp.* remain the most frequently detected pathogens in infections with local relation to the oral cavity and its bacterial flora [32, 33]. Likewise, *Streptococcus spp.* were the most frequently harvested bacteria within this study’s cohort. In early stages of oral wound infections, sacchrolytic bacteria, mainly *Streptococcus spp.*, promote an aciduric milieu, which in turn further promotes the growth of other aciduric bacteria, ultimately disturbing the healing process [29, 32]. Besides beta-hemolytic *Streptococcus spp.*, *P. aeruginosa* and *S. aureus* are also regarded as one of the primary causes of delayed wound healing in acute and chronic wounds [5, 32, 33]. In this study, *Streptococcus spp.*, *Prevotella spp*., and *Staphylococcus spp.* were most frequently harvested, as shown in Table 5. Among the facultative anaerobic gram-positive and gram-negative bacterial flora, *Pseudomonas aeruginosa* was less commonly detected, as displayed in Table 6, but significantly present in OAI of plates with greater volumes, as seen in Table 9. In contrast, *Streptococcus spp.*, *Prevotella spp*., and *Staphylococcus spp*. showed greater presence in OAI of plates with smaller volumes. Moreover, it is impossible to identify a singular pathogen responsible for OAI, as persistent wound infections are mediated by polymicrobial biofilms [4, 32, 34]. A multifaceted approach including local and systemic antibiotics, surgical debridement, adequate wound dressing, and optional hardware removal is required for the proper treatment of biofilm-based OAI [4]. Bacterially colonized osteosynthetic material with corresponding OAI is not likely to resolve spontaneously. Once a solid biofilm inhabited less by planktonic bacteria has been successfully established, persistent infection with continuous complications and inadequate levels of inflammation and immune response may occur, thus resulting in a state of chronic infection [4, 30]. Systemic antibiotics are useful for acute infections and for the prevention of the aforementioned [27, 33]. Chronic biofilms are nevertheless most efficiently treated with local antibiotics, as significantly higher doses without the associated risks of systemic toxicity may be applied [10]. However, successful management of higher-stage acute and chronic osteomyelitis generally requires a combination of surgical intervention and systemic antibiotic application [11].

**Table 9:**
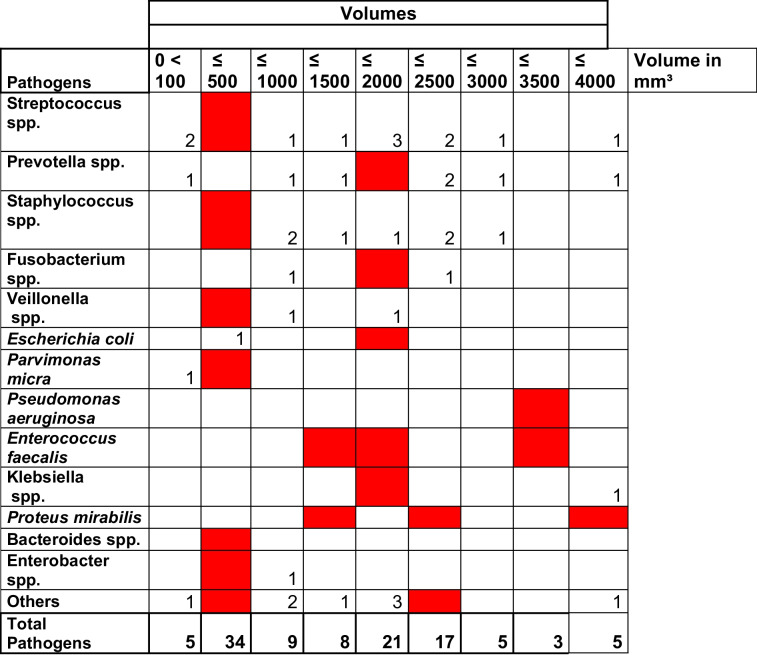
Antibiotics tested for susceptibility on more than 45 bacteria are bold printed

Cultivated bacteria are listed in line with the volumes of the colonized materials. The different Majority vol. are highlighted

Notably, this study is not only limited by its retrospective nature but also by the inability of microbial culture methods to identify the diversity of the oral microbiota. In order to determine bacterial diversity as a whole, high-throughput sequencing methods must be applied [17]. Moreover, a fully comprehensive picture of the diversity and composition of the oral microbiome requires molecular techniques such as DNA-DNA hybridization, polymerase chain reaction, and DNA sequencing [2].

Within this study’s microbial cultures, harvested pathogens expressed high susceptibility to systemically administered piperacillin/tazobactam, 3rd-generation cephalosporines, cefotaxime, and imipenem, as displayed in Table 9. Previous investigations have already highlighted the valuable benefits of piperacillin/tazobactam and 3rd-generation cephalosporines for the treatment of severe infections with oral biofilms [32, 34]. Our data nevertheless remains limited to recommended systemic antibiotic therapies and cannot predict the efficiency of topical antibiotic agents.

## Conclusion

Large-volume osteosynthetic materials and lower jaw reconstructions bear the greatest risks for OAI. For OAI of large volume implants, gram-negative pathogens must be targeted with an appropriate antibiotic regime, e.g., piperacillin/tazobactam and/or 3^rd^-generation cephalosporines.

## Data Availability

Data was fully available to all authors, accept FP. FP commented on the manuscript.
